# Curcumin Protects Human Trophoblast HTR8/SVneo Cells from H_2_O_2_-Induced Oxidative Stress by Activating Nrf2 Signaling Pathway

**DOI:** 10.3390/antiox9020121

**Published:** 2020-02-01

**Authors:** Lina Qi, Jingle Jiang, Jingfei Zhang, Lili Zhang, Tian Wang

**Affiliations:** National Experimental Teaching Demonstration Center of Animal Science, College of Animal Science and Technology, Nanjing Agricultural University, Nanjing 210095, China; 2018205023@njau.edu.cn (L.Q.); 2018205008@njau.edu.cn (J.J.); zhangjingfei@njau.edu.cn (J.Z.); zhanglili@njau.edu.cn (L.Z.)

**Keywords:** curcumin, HTR8/SVneo cells, Nrf2, oxidative stress

## Abstract

Pregnancy complications are associated with oxidative stress induced by accumulation of trophoblastic ROS in the placenta. We employed the human trophoblast HTR8/SVneo cell line to determine the effect of curcumin pre-treatment on H_2_O_2_-induced oxidative damage in HTR8/Sveo cells. Cells were pretreated with 2.5 or 5 μM curcumin for 24 h, and then incubated with 400 μM H_2_O_2_ for another 24 h. The results showed that H_2_O_2_ decreased the cell viability and induced excessive accumulation of reactive oxygen species (ROS) in HTR8/Sveo cells. Curcumin pre-treatment effectively protected HTR8/SVneo cells against oxidative stress-induced apoptosis via increasing Bcl-2/Bax ratio and decreasing the protein expression level of cleaved-caspase 3. Moreover, curcumin pre-treatment alleviated the excessive oxidative stress by enhancing the activity of antioxidative enzymes. The antioxidant effect of curcumin was achieved by activating Nrf2 and its downstream antioxidant proteins. In addition, knockdown of Nrf2 by Nrf2-siRNA transfection abolished the protective effects of curcumin on HTR8/SVneo cells against oxidative damage. Taken together, our results show that curcumin could protect HTR8/SVneo cells from H_2_O_2_-induced oxidative stress by activating Nrf2 signaling pathway.

## 1. Introduction

Intrauterine growth retardation (IUGR) and preeclampsia (PE) are detrimental pregnancy complications that could cause significant increased perinatal morbidity and mortality [1]. Normal placental development during early pregnancy depends entirely on the differentiation, proliferation, and invasion of trophoblast cells [2]. IUGR refers to impaired growth and development of the fetus or fetal organs, and these consequences are associated with the dysfunction of placental trophoblast cells [3].

Oxidative stress arose from the production of reactive oxygen species (ROS) reduces the antioxidant capacity of cells, which, in turn, results in cell damage and eventually cell death [4,5]. During normal pregnancy, the metabolisms of the mother and fetus are enhanced because of higher energy and oxygen requirements [6]. This could consequently accelerate the accumulation of ROS, and eventually induce excessive oxidative stress in the trophoblast cells. Nevertheless, cells have developed an antioxidant defense system to protect against oxidative stress. The system consists of antioxidant enzymes such as glutathione (GSH), glutathione peroxidase (GSH-Px), superoxide dismutase (SOD) and catalase (CAT), which could scavenge ROS to prevent possible cellular damage [7]. Previous studies have demonstrated that antioxidants could attenuate the oxidative damage via enhancing the activities of these antioxidant enzymes [8,9]. Thus, antioxidants might protect placental trophoblast cells from excessive oxidative stress during pregnancy. It is necessary to explore antioxidants with prophylactic or therapeutic potential for placental dysfunction.

Curcumin, a major constituent derived from the root of curcuma longa, has antioxidant, anti-inflammatory and antimicrobial properties [10,11]. Numerous studies have indicated that curcumin is an effective antioxidant both in vivo and in vitro [12,13,14]. Several studies have shown that curcumin treatment could attenuate cell apoptosis, decrease the level of lipid peroxidation, and increase the activity of SOD [15,16,17,18]. The underlying mechanism is associated with the function of NFE2-related factor-2 (Nrf2) [19]. Recent studies have suggested that Nrf2 plays some functionally significant roles in protecting against oxidative stress and apoptotic damage [20,21,22]. A previous study has implicated that curcumin treatment is able to up-regulate the expression of Nrf2, NADP(H) quinine oxidoreductase 1 (NQO1) and heme oxygenase-1 (HO-1) [17]. In addition, Woo et al. has found that curcumin protects retinal pigment epithelial cells against oxidative stress via increasing the expression of HO-1 [23]. Yu et al. has reported that the regulation of Nrf2/HO-1 pathway is vital for protecting placenta against oxidative stress [24]. Our recent study has shown that daily curcumin supplementation could improve maternal placental function and fetal growth in mice with IUGR [25]. However, data are lacking in elaborating the potential molecular mechanism of curcumin against placental dysfunction under oxidative stress. 

Therefore, we used human trophoblast HTR8/Sveo cell line as an in vitro model of placental trophoblast cells. The objective of this study was to investigate whether curcumin exerts antioxidant protection on HTR8/Sveo cells against H_2_O_2_-induced oxidative damage and to explore the potential molecular mechanism.

## 2. Materials and Methods

### 2.1. Chemicals and Reagents

Curcumin and dimethyl sulfoxide (DMSO) were purchased from Sigma Aldrich Chemicals (St. Louis, MO, USA). Phosphate-Buffered Saline (PBS), Dulbecco’s Modified Eagle’s Medium (DMEM/F-12), fetal bovine serum (FBS), trypsin and penicillin-streptomycin were purchased from Gibco, Invitrogen (Carlsbad, CA, USA).

### 2.2. Cell Culture

Human trophoblast HTR8/SVneo cell line was obtained from the FuHeng Cell Center (Shanghai, China). Cells were cultured in DMEM/F-12 supplemented with 10% fetal bovine serum and 1% penicillin-streptomycin in a humidified incubator containing 5% CO_2_ at 37 °C. Cells in the logarithmic growth phase were used in subsequent experimentation. Curcumin were dissolved in DMSO and stored at −20 °C. The final concentration of DMSO in the medium was kept less than 0.1%, which has been shown nonlethal to the cells [26]. Cells in the H_2_O_2_-treated group were treated with 400 μM H_2_O_2_ alone for 24 h. Cells in the curcumin + H_2_O_2_-treated groups were pre-treated with 2.5 μM or 5 μM curcumin for 24 h, respectively, and then they were treated with 400 μM H_2_O_2_ for another 24 h.

### 2.3. Cell Viability Assay

Cell viability was determined following treatment with curcumin and H_2_O_2_, with a Cell Counting kit-8 assay (Dojindo Molecular Technologies, Inc. Shanghai, China). HTR8/SVneo cells were cultured in 96-well culture plates for 24 h. Then, after different concentration of curcumin and H_2_O_2_ treatment for 24 h, a total of 10 μL CCK-8 reagent was added in and incubated for 2 h in a 5% CO_2_ incubator at 37 °C. Finally, the optical density values were acquired with a microplate reader at 450 nm.

### 2.4. Analysis of the Contents of CAT, GSH-Px and ROS

After experimental treatment, cells were lysed with RIPA lysis buffer (Beyotime Biotechnology, Shanghai, China) and centrifugated at 12,000× *g* for 10 min at 4 °C. Protein quantification was performed using a Bicinchoninic acid (BCA) protein assay kit (Beyotime Biotechnology, Shanghai, China). The activities of CAT, GSH-Px and the content of ROS were determined using the corresponding assay kits (Beyotime Biotechnology, Shanghai, China) according to the manufacturer’s instructions. All results were normalized to protein concentration in each sample.

### 2.5. Analysis of Apoptosis by Annexin V-Alexa Fluor 647/Propidium Iodide (PI) Staining

The cell apoptosis in each group treated with 2.5 or 5 μM curcumin for 24 h and 400 μM H_2_O_2_ for another 24 h were determined by an Annexin V-Alexa Fluor 647/propidium iodide double-stain assay, according to the manufacturer’s protocol (Annexin V-Alexa Fluor 647/PI Apoptosis Assay Kit, FMSAV647-100, FcMACS, Nanjing, China). Briefly, adherent cells (1 × 10^6^) of the four experimental groups were collected by trypsin and resuspended in 100 μL binding buffer containing 5 μL Annexin V/Alexa Fluor 647 and 10 μL 20 μg/mL PI for 15 min at room temperature in dark. The flow cytometric analyses were performed on flow cytometry (Becton Dickinson). FlowJo software was used for data acquisition and analysis.

### 2.6. Quantitative Real-Time PCR

Total RNA was isolated from the cells with TRIzol reagent (TaKaRa, Dalian, China) and processed for quantitative real-time PCR. Total RNA was reverse transcribed into cDNA with PrimeScript reverse transcriptase reagent kit (TaKaRa, Dalian, China). Real-time PCR analysis was performed using a QuantStudio 5 Real-Time PCR System (Thermo Scientific, Wilmington, USA) and a TB Premix Ex Taq Kit (TaKaRa, Dalian, China). The reaction program was as follows: 95 °C for 30 s, 40 cycles of 95 °C for 10 s and 60 °C for 30 s. The melting curve was used to verify the amplification of a single product. The primers were synthesized by Sangon Biotech (Sangon Biotech Co., Ltd., Shanghai, China), and the primer sequences used in this study were shown in Table 1. All samples were measured in triplicate, and the data were analyzed using the 2^−△△Ct^ method.

### 2.7. Nrf2 Immunofluorescence

The nuclear translocation of Nrf2 was determined by immunofluorescence. Briefly, HTR8/SVneo-cells were treated as previously described. Then, the cells were washed 3 times with PBS, fixed in 4% paraformaldehyde, permeabilized with 0.5% Triton X-100, blocked with 1% BSA and then incubated with anti-Nrf2 antibody (Proteintech, 16396-1-AP, Wuhan, China, 1:200 dilution) overnight at 4 °C. The bound antibody was detected by Cy3 conjugated Goat Anti-rabbit IgG (Servicebio, GB21303, Wuhan, China). The nuclei were stained with 4’,6-diamidino-2-phenylindole staining solution (C1005, Beyotime Biotechnology, Shanghai, China). The coverslips were inverted on glass slides and then examined under a Pannoramic 250 (3D HISTECH) using the same parameter settings among the different treated groups.

### 2.8. Western Blotting

Cells were washed twice with PBS, and then suspended in 100 μL RIPA (Beyotime Biotechnology, Shanghai, China) containing 1 mM phenylmethylsulfonyl fluoride (PMSF, Beyotime Biotechnology, Shanghai, China), followed by centrifugation at 12,000× *g* for 10 min at 4 °C. Nucleoprotein was extracted with Nuclear and Cytoplasmic Extraction Reagents (Thermo Scientific, Wilmington, DE, USA). The protein concentrations were measured by a BCA protein assay kit (Beyotime Biotechnology, Shanghai, China). The lysate (10 μg protein/lane) was resolved in 4%–20% SDS-PAGE and then transferred to PVDF membranes (Millipore, Bedford, MA, USA). After the membranes were blocked with 5% bovine serum albumin (BSA) for 2 h at room temperature, target proteins were immunodetected using specific antibodies. The membranes were incubated with specific primary antibodies against Nrf2, HO-1, NQO1, Bcl-2, Bax and Cleaved-Caspase 3 (Proteintech, 16396-1-AP; Zen-Bioscience, 380753; Proteintech, 11451-1-AP; Affinity, AF6139; Proteintech, 50599-2-lg; Affinity, AF7022) overnight at 4 °C. Histone H3 (Proteintech, 17168-1-AP) was applied as a loading control for nucleoprotein. β-Actin (Proteintech, 660091-1) was utilized as a loading control for total protein. After three washes in TBST for 10 min each, the membranes were incubated with secondary antibody (1:2000, AS003, ABclonal Biotechnology Co., Ltd., Wuhan, China) for 60 min at room temperature. Finally, the blots were washed in TBST for three times and protein bands were detected using an enhanced chemiluminescence (ECL) kit (Thermo Scientific, Wilmington, DE, USA). Signals were visualized using Luminescent Image Analyzer LAS4000 (FuJI Film, Tokyo, Japan). The protein expressions were estimated by quantifying the intensities of the bands using ImageJ software.

### 2.9. Small Interfering RNA (siRNA) Transfection

The human-specific siRNAs targeting Nrf2 were designed and synthesized by GenePharma (Shanghai, China). For transfection, the HTR8/SVneo cells were seeded in 6-well culture plates and siRNA-Nrf2 were transfected into the cells using the Lipofectamine™ 2000 Transfection reagent (Thermo Scientific, Wilmington, DE, USA) prior to treatment with curcumin and H_2_O_2_ according to the manufacturer’s instructions. The target sequences used in this study were shown in Table 2.

### 2.10. Statistical Analysis

All the data are presented as mean ± SEM for at least three independent experiments. The Shapiro–Wilk test was used to estimate the normality distribution of the data. Statistical differences were determined by one-way ANOVA followed by Tukey’s test (Graph Pad Software Inc. San Diego, CA, USA). Differences were considered to be significant at *p* < 0.05.

## 3. Results

### 3.1. Curcumin Protected against H_2_O_2_-Induced Cytotoxicity in HTR8/SVneo Cells

**Figure 1:**
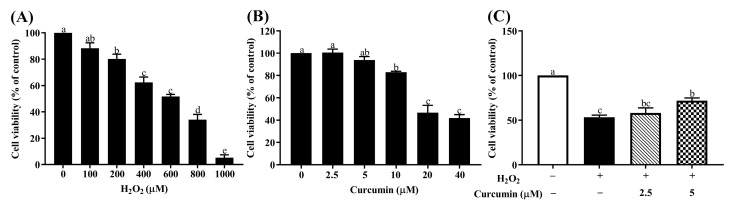
Effects of curcumin on H_2_O_2_-induced cell viability in HTR8/SVneo cells measured by the CCK-8 assay; (**A**) Cell viability of HTR8/SVneo cells treated with different concentrations of H_2_O_2_; (**B**) cell viability of HTR8/SVneo cells treated with different concentrations of curcumin; (**C**) cell viability of HTR8/SVneo cells pretreated with 2.5 or 5 μM curcumin against H_2_O_2_-induced oxidative stress. Data are presented as the mean ± SEM (*n* = 3) ^a, b, c^ Means with different letters are significantly different (*p* < 0.05).

Firstly, to achieve the optimized oxidative stress conditions, we examined HTR8/SVneo cells treated with different concentrations of H_2_O_2_ (100, 200, 400, 600, 800 and 1000 μM) for 24 h. Cell viabilities were analyzed using a CCK-8 assay. As shown in Figure 1A, a dose-dependent increase in cytotoxicity of HTR8/SVneo cells was observed in response to H_2_O_2_. The IC50 value of H_2_O_2_ concentration was 400 μM which resulted in 50% inhibition of HTR8/SVneo cells. Thus, 400 μM H_2_O_2_ treatment for 24 h was chosen to perform subsequent experiments. Secondly, to evaluate cell viability in different concentrations of curcumin and to determine its non-cytotoxic concentration, HTR8/SVneo cells were pretreated with different concentrations of curcumin (0, 2.5, 5, 10, 20 and 40 μM) for 24 h. As shown in Figure 1B, 10 μM curcumin has cytotoxicity to HTR8/SVneo cells, thus we chose 2.5 and 5 μM curcumin for the following experiments. Finally, to assess the cytoprotective effect of curcumin, HTR8/SVneo cells were pretreated with 2.5 and 5 μM curcumin for 24 h, followed by 400 μM H_2_O_2_ for another 24 h. As shown in Figure 1C, the 5 μM curcumin pretreatment group significantly increased the cell viability was significantly increased in the 5 μM curcumin pre-treatment group compared with the H_2_O_2_ treatment group.

### 3.2. Curcumin Increased H_2_O_2_-Induced CAT, GSH-Px Activity and Reduced the Level of Intracellular ROS in HTR8/SVneo Cells under Oxidative Stress

**Figure 2:**
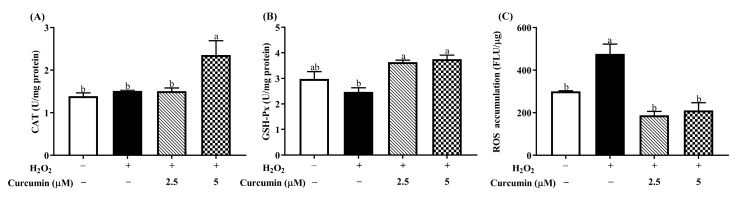
Effect of curcumin on the activity of antioxidant enzymes and ROS generation. Cells were pretreated with 2.5 or 5 μM curcumin for 24 h, and then incubated with 400 μM H_2_O_2_ for another 24 h. The activity of cellular CAT (**A**), GSH-Px (**B**) and ROS accumulation (**C**) were measured. ROS production was determined using the DCFH-DA assay. Data are presented as the mean ± SEM (*n* = 3). ^a, b^ Means with different letters are significantly different (*p* < 0.05).

To evaluate the effects of curcumin on the activity of antioxidant enzymes in HTR8/SVneo cells, the activities of CAT and GSH-Px were assessed. As shown in Figure 2A,B, the activities of CAT and GSH-Px showed no significant difference (*p* > 0.05) between the control and H_2_O_2_ treatment group. Whereas, pre-treatment with 5 μM curcumin followed by 400 μM H_2_O_2_ up-regulated (*p* < 0.05) the activities of CAT and GSH-Px in HTR8/SVneo cells. To further prove the protective of curcumin against oxidative stress, we measured the intracellular level of ROS by using fluorescent probe DCFH-DA. As shown in Figure 2C, the accumulation of ROS in HTR8/SVneo cells was significantly increased (*p* < 0.05) in the H_2_O_2_ group compared with the control group, while pre-treatment with 2.5 and 5 μM curcumin remarkably reduced (*p* < 0.05) the H_2_O_2_-induced ROS accumulation in HTR8/SVneo cells.

### 3.3. Curcumin Inhibited H_2_O_2_-Induced Apoptosis of HTR8/SVneo Cells

**Figure 3:**
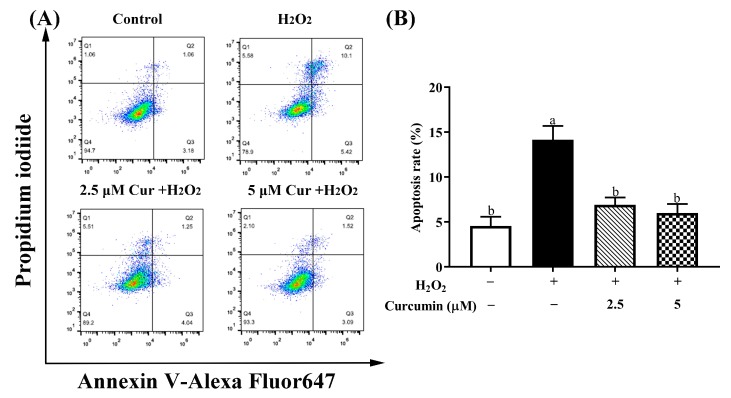
Effects of curcumin on H_2_O_2_-induced apoptosis in HTR8/SVneo cells measured by flow cytometry. (**A**) Control, the control group; H_2_O_2_, H_2_O_2_ treatment group; 2.5 μM Cur + H_2_O_2_, 2.5 μM curcumin pre-treatment followed by the treatment of 400 μM H_2_O_2_; 5 μM Cur + H_2_O_2_, 5 μM curcumin pre-treatment followed by the treatment of 400 μM H_2_O_2_. (**B**) The apoptosis rates of HTR8/SVneo cells after different treatments. Data are presented as the mean ± SEM (*n* = 3). ^a, b^ Means with different letters are significantly different (*p* < 0.05).

To further evaluate the inhibitory effect of curcumin on H_2_O_2_-induced apoptosis in HTR8/SVneo cells, apoptotic rates were measured by Annexin V-FITC/PI double staining using flow cytometry. As shown in Figure 3, the percentage of apoptotic cells was 4.56% ± 1.04% in the control group, whereas that of the H_2_O_2_ treatment group was 14.13% ± 1.56% (*p* < 0.05). However, pre-treatment with 5 μM curcumin markedly decreased apoptotic rates to 6.00% ± 1.00% (*p* < 0.05) in HTR8/SVneo cells treated with 400 μM H_2_O_2_. 

### 3.4. Curcumin Regulated mRNA Expression of Multiple Antioxidant Genes and Nutrient Transporter Genes in HTR8/SVneo Cells under Oxidative Stress

**Figure 4:**
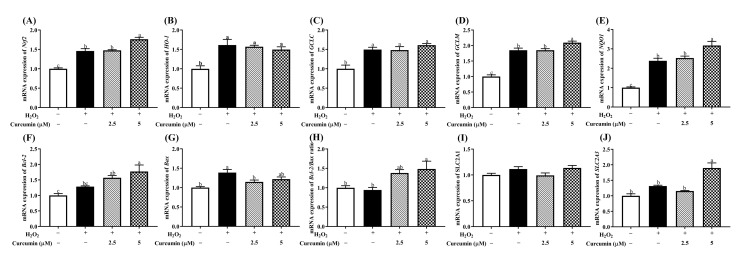
Effects of curcumin on the mRNA expression of *Nrf2* (**A**)*, HO-1* (**B**)*, GCLC* (**C**)*, GCLM* (**D**)*, NQO1* (**E**)*, Bcl-2* (**F**)*, Bax* (**G**)*, Bcl-2/Bax* ratio (**H**)*,* *SLC2A1* (**I**) and *SLC2A3* (**J**) gene expression. Cells were pre-treated with 2.5 or 5 μM curcumin for 24 h, and then treated with 400 μM H_2_O_2_ for another 24 h. GAPDH was used as a housekeeping gene. Data are presented as the mean ± SEM (*n* = 3). ^a, b, c^ Means with different letters are significantly different (*p* < 0.05). No letters or the same letters mean the no significantly difference.

Since curcumin has been shown to exert antioxidant effect by inducing several antioxidant defense enzymes in several cell lines [14,23]. Thus, we performed RT-qPCR to detect whether the protective function of curcumin against the oxidative stress induced by H_2_O_2_ was associated with antioxidant-associated factors in HTR8/SVneo cells. Our results showed that H_2_O_2_ stimulation alone significantly increased the mRNA levels of *Nrf2, HO-1*, *GCLC, GCLM* and *NQO1* (*p* < 0.05), whereas pre-treatment of 5 μM curcumin further up-regulated (*p* < 0.05) the expression of *Nrf2*, *GCLM* and *NQO1*. H_2_O_2_ treatment alone increased (*p* < 0.05) the transcription level of *Bax*, and pre-treatment with 5 μM curcumin increased (*p* < 0.05) the expression of *Bcl-2*. The expression of *Bcl-2/Bax* was also up-regulated (*p* < 0.05) in both curcumin pre-treatment groups (Figure 4F–H). In addition, H_2_O_2_ treatment alone had no effect (*p* > 0.05) on the mRNA expression of solute carrier family 2 member 1 (*SLC2A1*) and solute carrier family 2 member 3 (*SLC2A3*), while pre-treatment with 5 μM curcumin increased (*p* < 0.05) the gene expression of *SLC2A3* (Figure 4I,J).

### 3.5. Curcumin Increased Nrf2, HO-1 and NQO1 Protein Expression and Nrf2 Translocation in HTR8/SVneo Cells under Oxidative Stress

**Figure 5:**
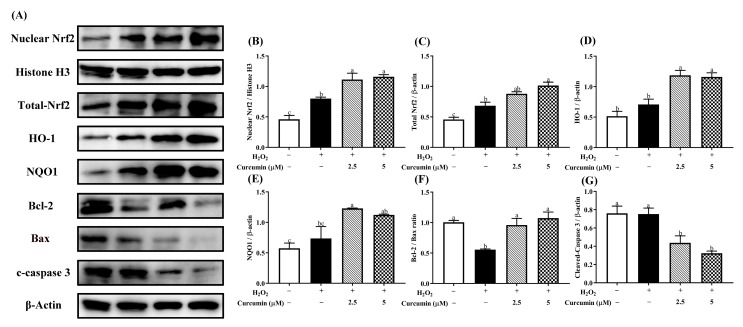
Effects of curcumin on nuclear Nrf2, total-Nrf2, HO-1, NQO1, Bcl-2, Bax and cleaved-caspase 3 protein expression. The bands (**A**) and relative protein expression of nuclear Nrf2 (**B**), total-Nrf2 (**C**), HO-1 (**D**), NQO1 (**E**), Bcl-2/Bax (**F**) and cleaved-caspase 3 (**G**) were measured by Western blotting. Cells were pre-treated with 2.5 or 5 μM curcumin for 24 h, and then treated with 400 μM H_2_O_2_ for another 24 h. Histone H3 was used as a loading control for nucleoprotein. β-actin was used as a loading control for total protein. Data are presented as the mean ± SEM (*n* = 3). ^a, b, c^ Means with different letters are significantly different (*p* < 0.05).

**Figure 6:**
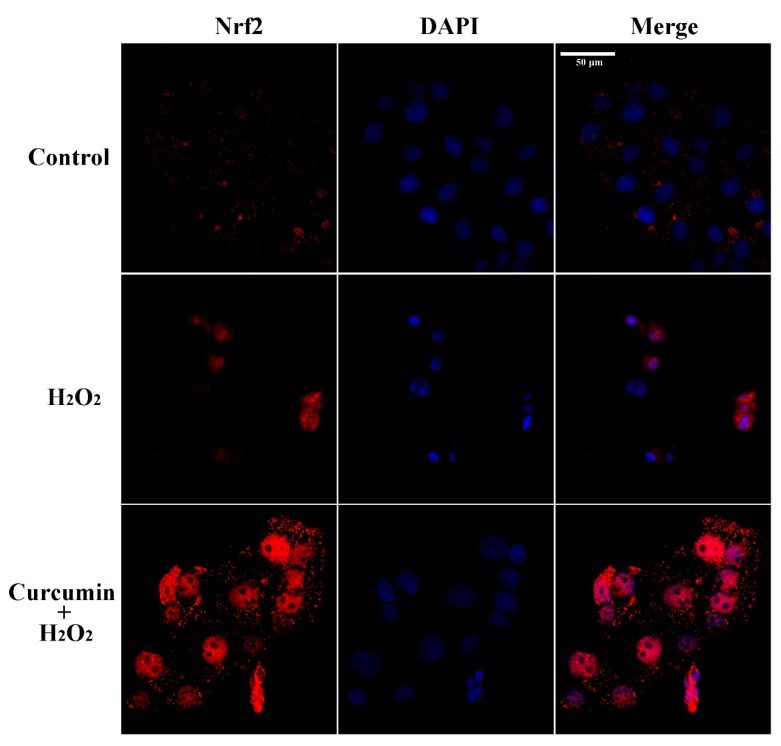
Effects of curcumin on Nrf2 nuclear translocation in HTR8/SVneo cells. Immunofluorescence staining of Nrf2 in HTR8/SVneo cells treated with H_2_O_2_ or pre-treated with 5 μM curcumin for 24 h and H_2_O_2_ for another 24 h. Blue and red colors indicate localization of DAPI (nuclei) and Nrf2, respectively. Scale bar = 50 μm.

As shown in Figure 5, H_2_O_2_ stimulation alone up-regulated (*p* < 0.05) the protein expression of Nrf2 and NQO1, while it did not affect (*p* > 0.05) the protein expression of HO-1. Pre-treatment with 2.5 or 5 μM curcumin up-regulated (*p* < 0.05) the protein expression of nuclear Nrf2, total-Nrf2, HO-1 and NQO1. In addition, immunofluorescence staining showed that both H_2_O_2_ and curcumin stimulated Nrf2 nuclear translocation in HTR8/SVneo cells (Figure 6). However, we observed that pre-treatment with curcumin induced more Nrf2 nuclear translocation compared with the H_2_O_2_ treatment group. Furthermore, pre-treatment with curcumin significantly increased the Bcl-2/Bax ratio and decreased the expression of active caspase-3 in H_2_O_2_-treated HTR8/SVneo cells (Figure 5A,E,F).

### 3.6. Nrf2 Knockdown Attenuated the Protective Effect of Curcumin on HTR8/SVneo Cells under Oxidative Stress

**Figure 7:**
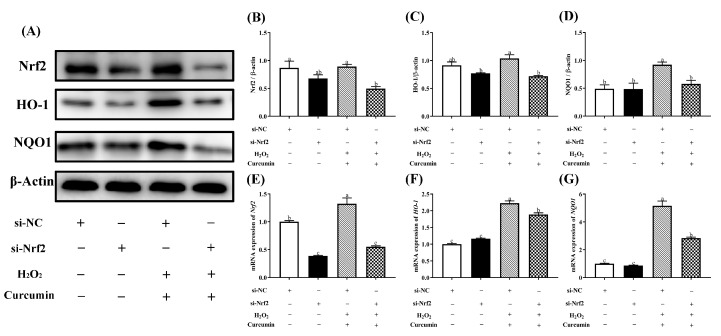
Silencing Nrf2 gene abolished the prevention made by curcumin. HTR8/SVneo cells transfected with siRNA against Nrf2 or control siRNA for 24 hours, followed by 24 h pre-treatment with 5 μM curcumin, and then treated with 400 μM H_2_O_2_ for another 24 h. The bands (**A**) and relative protein expression of Nrf2 (**B**), HO-1 (**C**) and NQO1 (**D**) were measured by Western blotting. The mRNA expression of *Nrf2* (**E**), *HO-1* (**F**) and *NQO1* (**G**) were measured by RT-PCR. Data are presented as the mean ± SEM (*n* = 3). ^a, b, c^ Means with different letters are significantly different (*p* < 0.05).

**Figure 8:**
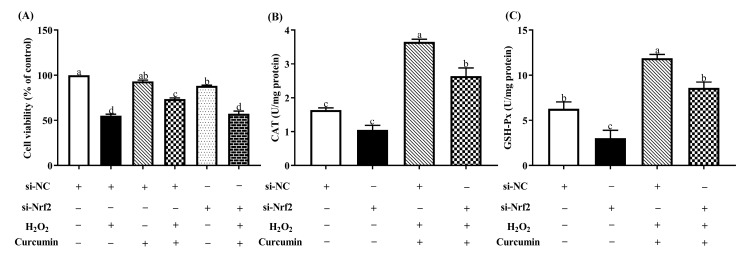
Silencing Nrf2 gene abolished the prevention made by curcumin. HTR8/SVneo cells transfected with siRNA against Nrf2 or control siRNA for 24 hours, followed by 24 h pre-treatment with 5 μM curcumin, and then treated with 400 μM H_2_O_2_ for another 24 h. The cell viability (**A**), CAT (**B**) and GSH-Px (**C**) activity of cells were determined respectively. Data are presented as the mean ± SEM (n=3). ^a, b, c, d^ Means with different letters are significantly different (*p* < 0.05).

To further elucidate the role of Nrf2 in the cytoprotective effects of curcumin against oxidative stress, we transfected HTR8/SVneo cells with a Nrf2 siRNA for 24 h, then pre-treated with 5 μM curcumin followed by the treatment of 400 μM H_2_O_2._ As shown in Figure 7, after the transfection with si-Nrf2 in curcumin and H_2_O_2_ treatment group, we observed markedly decreased (*p* < 0.05) mRNA and protein expression level of Nrf2, HO-1 and NQO1 compared with the curcumin + H_2_O_2_ transfected with si-NC group. In addition, Nrf2 silencing abolished (*p* < 0.05) the upregulation of cell viability caused by curcumin pretreatment (Figure 8A). The activation of CAT and GSH-Px were significantly decreased (*p* < 0.05) with knockdown of Nrf2 compared with the curcumin + H_2_O_2_ transfected with si-NC group (Figure 8C). 

## 4. Discussion

Placental trophoblast cells play important roles in the pregnancy and the development of the fetus. Excessive accumulation of trophoblastic ROS induced by greater maternal and fetal metabolism has been considered as an important factor that leads to placental dysfunction [27]. In the present study, we found for the first time that curcumin, a natural antioxidant known for its cytoprotective and anti-apoptotic actions [28,29], ameliorates H_2_O_2_-induced oxidative stress and cell apoptosis in human trophoblast HTR8/SVneo cells by activating the Nrf2 signaling pathway.

It is well established that H_2_O_2_ could be used to stimulate oxidative stress in vitro [30,31,32]. In the present study, we found that treatment with 400 μM H_2_O_2_ resulted in decreased cell viability in HTR8/SVneo cells, which is consistent with a previous study [1]. Curcumin has been reported to be toxic at a high dose, while it exerts a strong antioxidant effect at a low dose [23]. In the present study, pre-treatment of more than 10 μM curcumin for 24 h can induce obvious cell death in HTR8/SVneo cells, so we chose 2.5 and 5 μM curcumin as the optimal concentrations of curcumin for the subsequent experiments. Similar with the previous findings, pre-treatment with curcumin had the ability to enhance the viability of HTR8/SVneo cells after treatment of H_2_O_2_ [23,33]. Moreover, the result of 5 μM curcumin pre-treatment showed a better effect than 2.5 μM, suggesting curcumin might exert the protective effect in a dose-dependent manner in HTR8/SVneo cells. These results indicate that low dose curcumin could protect HTR8/SVneo cells from death induced by H_2_O_2_.

ROS are oxygen free radicals, and the accumulation of ROS could cause oxidative stress and cell apoptosis [33]. Excessive oxidative stress induced by trophoblastic ROS is a pivotal factor for IUGR [27]. Our data showed that the exposure of HTR8/Sveo cells to H_2_O_2_ resulted in increased intracellular ROS accumulation, but it was significantly alleviated by pre-treatment with either 2.5 μM or 5 μM curcumin. Consistent with our result, a previous study has also found that curcumin is able to reduce ROS accumulation [34]. This effect might be related with augmented antioxidant defense system promoted by curcumin pre-treatment. In addition, CAT and GSH-Px are the main members of antioxidant defense system. CAT converts hydrogen peroxide into water and oxygen [35]. GSH-Px is a vital antioxidant enzyme that catalyzes the reduction of hydroperoxides at the expense of reduced GSH [36]. In agreement with a previous finding that curcumin could improve the antioxidant capacity, curcumin pre-treatment enhanced the activities of CAT and GSH-Px in the HTR8/Sveo cells treated with H_2_O_2_ [37]. The antioxidant property of curcumin has been reported to be mainly associated with its free radical scavenging activity [15]. Thus, our results indicate that pre-treatment with curcumin could reduce oxidative stress in HTR8/Sveo cells treated with H_2_O_2_.

Oxidative stress can induce excessive cell apoptosis through either mitochondria-dependent or independent pathway [38]. Equally, the apoptosis rate can also reflect the degree of oxidative stress [39]. We observed that apoptosis was sharply promoted in the H_2_O_2_ treatment group. Supportively, H_2_O_2_ treatment reduced the protein expression level of Bcl-2/Bax ratio. Bcl-2 is the core molecule which plays a considerable role of resistance in apoptosis, whereas Bax is a promoting apoptosis protein. Bcl-2/Bax ratio has been reported to be an important determinant factor of apoptosis [40]. Ample pieces of evidence have also proven that H_2_O_2_ treatment could increase cell apoptosis [32,41]. However, pre-treatment with curcumin inhibited cell apoptosis, which was demonstrated by declined apoptosis rate, reduced protein expression of cleaved-caspase 3 and increased expression level of Bcl-2/Bax ratio. These results were consistent with the previous studies, which have also affirmed the anti-apoptotic effects of curcumin against oxidative stress-induced apoptosis [42]. Moreover, apoptosis is an essential regulatory cell process that occurs via caspase-independent pathway or caspase-dependent pathway [43]. Therefore, the decline of cleaved-caspase 3 protein expression level suggests that pre-treatment with curcumin can suppress apoptosis in HTR8/Sveo cells through inhibiting caspase-dependent pathway. Further functional investigation should be performed to estimate whether curcumin could reduce apoptosis in trophoblast cells via inhibiting caspase-independent pathway.

Nrf2 is a vital transcription factor that regulates cell survival and maintains redox homeostasis [44,45]. Nrf2 signaling pathway is also a crucial antioxidant pathway, which is responsible for regulating the expression of antioxidant enzymes against oxidative stress in cells [46,47,48]. The transcription of *HO-1* and *NQO1*, the downstream genes of Nrf2, are primarily under the control of Nrf2 in maintaining cell redox balance [49,50]. Under oxidative stress, Nrf2 could translocate into the nucleus to activate the expression of its downstream antioxidant enzymes [32]. Similar with the previous studies, the Nrf2 signaling was activated by H_2_O_2_ treatment in order to compensate for the oxidative damage [32,51]. Nrf2 nuclear translocation was also observed in the H_2_O_2_ treatment group. In addition, previous researchers have made great effort to prove that curcumin activates Nrf2 and HO-1 signaling leading to protection against oxidative stress in different cell types [17,19,52,53]. In accordance with these findings, pre-treatment with curcumin induced a more obvious Nrf2 nuclear translocation than the H_2_O_2_ treatment alone. Combined with the results of more effectively enhanced mRNA and protein expression of Nrf2, HO-1 and NQO1 induced by pre-treatment with curcumin, our data suggested that curcumin exerted its antioxidant effect against oxidative stress in HTR8/SVneo cells by promoting the activation of Nrf2 signaling pathway. The enhanced activation of Nrf2 signaling could also explain for the increased activity of CAT and decreased accumulation of ROS. In addition, up-regulation of nutrient transporters (*SLC2A3*) can improve the transfer of nutrients (including amino acids, fatty acids, and glucose) from mother to fetus, which plays an important role in placental development and fetal growth [54,55]. In our previous in vivo experiment, we have proven that curcumin has beneficial effects on nutrient transport and placental development, which could be applied for alleviating IUGR of mice. Furthermore, we found that knockdown of Nrf2 by Nrf2-siRNA transfection markedly diminished the curcumin-induced up-regulation of Nrf2, HO-1 and NQO1. Similarly, a previous study has found that knockdown of Nrf2 could decrease the expression level of HO-1 and NQO1 [56]. Here, we have shown that knockdown of Nrf2 decreased the protective effect of curcumin pre-treatment on the cell viability against oxidative stress, suggesting that Nrf2 is a key factor in maintaining the survival of HTR8/SVneo cells. Since Nrf2 controls the transcription of antioxidant enzymes, the increased activities of CAT and GSH-Px by pre-treatment with curcumin were also reduced after knockdown of Nrf2. Collectively, our data strongly proves that the antioxidant effect of curcumin on HTR8/SVneo cells against oxidative stress is achieved by activation of Nrf2 signaling pathway.

## 5. Conclusions

In conclusions, this is the first study demonstrating that pre-treatment with curcumin could alleviate H_2_O_2_-induced oxidative stress in human trophoblast HTR8/SVneo cells by improving the antioxidant capacity and activating Nrf2 signaling pathway.

## Figures and Tables

**Table 1:** The primer sequences for real-time PCR.

Genes (GenBank)	Primer Sequences (5’-3’)	Product Size (bp)
*N* *rf* *2*	F: CTTGGCCTCAGTGATTCTGAAGTG	124
(NM_006164.5)	R: CCTGAGATGGTGACAAGGGTTGTA	
*HO-1*	F: CAGGAGCTGCTGACCCATGA	195
(NM_002133.3)	R: AGCAACTGTCGCCACCAGAA	
*GCLC*	F: GAAGTGGATGTGGACACCAGATG	128
(NM_001498.4)	R: TTGTAGTCAGGATGGTTTGCGATAA	
*GCLM*	F: GGAGTTCCCAAATCAACCCAGA	71
(NM_002061.4)	R: TGCATGAGATACAGTGCATTCCAA	
*NQO1*	F: GGATTGGACCGAGCTGGAA	140
(NM_000903.3)	R: AATTGCAGTGAAGATGAAGGCAAC	
*Bcl-2*	F: ATAACGGAGGCTGGGTAGGT	127
(NM_000657.2)	R: TTTATTTCGCCGGCTCCACA	
*Bax*	F: GCCCTTTTGCTTCAGGGGATG	76
(NM_138763.4)	R: CAGCTGCCACTCGGAAAAAG	
*SLC2A1*	F: TGAGCATCGTGGCCATCTTT	298
(NM_006516.3)	R: CCGGAAGCGATCTCATCGAA	
*SLC2A3*(NM_006931.3)	F: GCACATAGCTATCAAGTGTGCTT R: CCTGCCTTACTGCCAACCTA	97
*GAPDH*	F: GACAGTCAGCCGCATCTTCT	104
(NM_002046.7)	R: GCGCCCAATACGACCAAATC	

*Nrf2*, nuclear factor-erythroid 2-related factor 2; *HO-1*, home oxygenase-1; *GCLC*, glutamate-cysteine ligase catalytic; *GCLM*, glutamate-cysteine ligase modifier; *NQO1*, NAD(P)H quinone dehydrogenase 1; *Bcl-2*, *Bcl-2* apoptosis regulator; *Bax*, *Bcl-2* associated X, apoptosis regulator; *SLC2A1*, solute carrier family 2 member 1; *SLC2A3*, solute carrier family 2 member 3; *GAPDH*, glyceraldehyde 3-phosphate dehydrogenase.

**Table 2:** Primer sequences of Nrf2 siRNA.

Name	Primer Sequences (5–3 Orientation)
siRNA-Nrf2	Sense: GGUUGAGACUACCAUGGUUTT Antisense: AACCAUGGUAGUCUCAACCTT
siRNA-NC	Sense: UUCUCCGAACGUGUCACGUTT Antisense: ACGUGACACGUUCGGAGAATT
